# Smoking relapse and withdrawal symptoms among smokers accessing smoking cessation services provided by the primary care settings of Qatar

**DOI:** 10.1177/22799036251401951

**Published:** 2025-12-04

**Authors:** Abduljaleel Abdullatif Zainel, Hanan Al Mujalli, Ameena Ibrahim Yfakhroo, Shaikha Abdulla Shaheen, Hafiz Ahmed E. Mohamed, Ahmed Sameer Al Nuaimi, Muslim Abbas Syed, Mohamed Ahmed Syed

**Affiliations:** 1 Department of Clinical Research, Primary Health Care Corporation, Doha, Qatar; 2 Primary Health Care Corporation, Doha, Qatar; 3 Department of Operation, Primary Health Care Corporation, Doha, Qatar

**Keywords:** smoking, cessation, relapse, withdrawal symptoms, primary care

## Abstract

**Objectives:**

To investigate and highlight the factors associated with smoking relapse and to demonstrate the withdrawal symptoms related to smoking cessation.

**Methods:**

This study is part of a larger historical cohort involved 490 participants who attended smoking cessation clinics in primary health care settings. A total of 143 relapsed after they initially quit smoking and included in this study. The participants were interviewed by phone using a structured questionnaire after obtaining informed consent. Frequency distribution tables and proportions were used to describe the study results.

**Results:**

More than half (55.9%) of participants who initially quit smoking by attending the smoking cessation clinics reported that relationships with smokers were the main reason for smoking relapse. Emotional or social problems led to relapse among 38.5% of the participants. Withdrawal symptoms were relatively low (14.7%). An increase in appetite was prevalent among 74.8% of smoking quitters as the main withdrawal symptom. This was followed by weight gain in 72%, craving for cigarettes/smoking in 71.3%, and feelings of anger in 53.1%. All the tested sociodemographic variables, namely: age, sex, nationality, income, education, and marital status, failed to show a statistically significant association with early timing of relapse (1–6 months).

**Conclusion:**

The study identified various factors linked to smoking relapse among individuals using smoking cessation services. Relationships with smokers and emotional or social problems were the most common reasons. Additional research is needed to investigate strategies and interventions specifically targeting early smoking relapse to attain the desired health outcomes from smoking cessation services.

## Introduction

Worldwide, smoking is the leading cause of preventable diseases, disability, and mortality.^1–3^ According to the World Health Organization (WHO), 1.3 billion people use tobacco products globally, resulting in 8 million deaths annually.^4^ Therefore, reducing tobacco use remains of utmost importance to protect lives and lessen the global disease burden.^5^

Various interventions and preventive strategies have been implemented globally to control tobacco use.^6,7^ These interventions mainly consist of therapeutic and behavioral measures.^6,7^ Clinician involvement increases the likelihood of smoking cessation. The goal of smoking cessation establishments is to offer smokers evidence-based help to quit.^8^ Tobacco dependence interventions, if delivered in a timely and effective manner, significantly decrease the smoker’s risk of developing smoking-related diseases.^9,10^ The WHO launched the Framework Convention on Tobacco Control (FCTC) as an internationally coordinated response to tackle the tobacco epidemic.^11^

Despite the perceived benefits of smoking cessation, many smokers who attempt to quit smoking experience relapse. The term relapse in this context refers to returning to smoking after quitting. It is important to note that relapsing is not a sign of failure, but rather an obstacle on the road to success.^12^ Most smokers quit and relapse several times before they finally achieve sustainable cessation.^13,14^ Previous studies indicate that smoking relapse is more frequent within a short time of cessation, mostly during the first weeks, with nearly 75% of smokers relapsing within 6 months.^13,15^ Moreover, the likelihood of relapse decreases with extended abstinence.^16,17^ In this context, a longer period of abstinence predicts a lower relapse rate among quitters.^15–17^ Literature indicates that various factors, including individual, interpersonal, and organizational influences, affect smoking relapse.^18^ Perceiving any negative impact on stress coping among quitters elevates the likelihood of smoking relapse. Developing specific coping strategies and highlighting the positive impacts of smoking cessation can help protect against relapse.^13^ In this context, following specific strategies and interventions such as intensive intervention, telephone counseling, and use of pharmacotherapy may contribute significantly in minimizing smoking relapse.^19,20^ The literature discusses many specific reasons for smoking relapse, including but not limited to living with other smokers,^21,22^ changes in emotional status due to smoking cessation,^23^ social problems,^24^ withdrawal symptoms,^25–30^ economic issues,^31^ and pharmaceutical therapy-related problems.^32,33^

This article represents a part of a larger study discussed the socio-demographic characteristics and smoking cessation prevalence among smokers attended primary care settings in Qatar. The study found that 63.5% of the study’s participants initially quit smoking after receiving smoking cessation services (SCSs).^17^ This section focuses relapse timing and the main reported reasons for smoking relapse among individuals who quit but later resumed smoking.

This study aimed to investigate the most common reasons leading to smoking relapse and to demonstrate the withdrawal symptoms associated with relapsing among attendees using the SCSs provided through the primary care settings of Qatar.

## Methods

### Study design

This study is a secondary data cross-sectional analysis of a larger historic cohort study conducted in the public primary health care system of Qatar.^17^ The original study employed survival analysis on a total of 490 smokers who used the smoking cessation clinic services in PHCC. That study reported on the effectiveness (success) of the smoking cessation program using the following follow-up periods: 1, 6, 12, 24, 36, and 42 months of quitting smoking during clinic visits. The current study reports on smoking relapse among those who initially quit smoking during their visit to the clinics. The reporting of this study conforms to the STROBE Checklist for strengthening the reporting of observational studies in epidemiology^34^ (added as a Supplemental File).

### Study population

Smokers who were accessing smoking cessation clinics and were registered at primary health care centers in Qatar on June 30th, 2021, were recruited for this study. Smokers who did not complete 42 months of follow-up since quitting smoking were excluded from the study.

### Data collection

A simple random sampling technique was used to extract contact details of 790 participants who attended the smoking cessation clinics (SCCs) from the Primary Health Care Corporation (PHCC) electronic registry after obtaining ethical approval from PHCC-IRB (PHCC/DCR/2019/10/028). Of those participants, only 490 agreed to participate in the study (of the 490 participants who agreed to join the study, 143 relapsed after quitting smoking and were included as the population of the current study). Data were collected through phone interviews with the participants using a structured questionnaire form between July and November 2022. The data collectors received initial training on delivering the phone interview in standardized way. Prior to data collection, the questionnaire was piloted amongst 20 selected past users of SCC, and their feedback was used to make any necessary amendments.

### The study tool

The researchers developed a structured questionnaire. An extensive literature review and consultation with the community medicine faculty and experts in the field of smoking cessation were used to assess the content and face validity of the tool. The questionnaire was originally prepared in English and translated into Arabic, with back translation to ensure its validity and reliability. The translation was done by the lead researcher using several medical dictionaries and then revalidated by two community medicine experts after reverse translation. After conducting an extensive literature review, the aim and objectives of the study served as a guide in developing the content of the questionnaire. An additional copy of the questionnaire was added as a Supplemental File.

Study variable included: sociodemographic, selected withdrawal symptoms of smoking cessation, selected reported reasons for smoking relapses, and relapse timing. The outcome variable of interest was the early relapse status which is defined as resuming the habit of smoking within the first 6 months of initial cessation.

### Data analysis

The Statistical Package for the Social Sciences (IBM SPSS V.28) was used for data entry and analysis. Descriptive analysis was conducted (frequency distribution tables and proportions) to assess the reported reasons for smoking relapse and selected smoking withdrawal symptoms. Analytical study performed to assess the factors associated with early smoking relapse. The chi-square test of independence was used to assess the statistical significance of associations.

## Results

The study sample was composed of a total of 143 study participants who initially quit smoking and relapsed afterward during the study period. Table 1 describes the sociodemographic characteristics of the study group.

**Table 1.** Frequency distribution of the study sample by sociodemographic variables (*N* = 143).

The variable	*N*	%
Age group (years)		
<30	12	8.4
30–39	65	45.5
40–49	39	27.3
50–59	19	13.3
60+	8	5.6
Total	143	100.0
Gender		
Female	7	4.9
Male	136	95.1
Total	143	100.0
Nationality		
Qataris	8	5.6
Arabs (excluding Qataris)	120	83.9
Others	15	10.5
Total	143	100.0
Average monthly income		
<10,000	73	51.0
10,000–19,999	42	29.4
≥20,000	28	19.6
Total	143	100.0
Ever married		
Single	16	11.2
Ever married	127	88.8
Total	143	100.0
Educational level		
Did not complete secondary school	28	19.6
Completed secondary school	21	14.7
Diploma/university education and above	94	65.7
Total	143	100.0

Among the most frequently reported withdrawal symptoms were the following three “Increase in appetite, weight gaining, and Craving for cigarettes/smoking.” These were reported in slightly less than three quarters of the study sample, Table 2.

**Table 2.** The relative frequency of selected smoking withdrawal symptoms (*N* = 143).

The symptom	*N*	%	95% confidence interval %
Increasing appetite	107	74.8	(67.3–81.4)
Weight gaining	103	72.0	(64.3–78.9)
Craving for cigarettes/smoking	102	71.3	(63.5–78.3)
Feeling of anger	76	53.1	(45–61.2)
Difficulty concentrating	61	42.7	(34.8–50.8)
Insomnia	45	31.5	(24.3–39.4)
Depressed mood	41	28.7	(21.7–36.5)
Increase in cough	38	26.6	(19.9–34.2)
Feeling of frustration	33	23.1	(16.8–30.5)
Developing of mouth ulcers	7	4.9	(2.2–9.4)
Weight losing	4	2.8	(1–6.5)

The two most important reasons for smoking relapse were “relationships with smokers (peer pressure) reported by more than a half (55.9%), and emotional/social problems” reported by 38.5% of the study sample, Table 3.

**Table 3.** The relative frequency of reported reasons for smoking relapse among those who initially quit smoking by attending the SCCs (*N* = 143).

Reported reason for smoking relapse	*N*	%	95% confidence interval %
Relationships with smokers	80	55.9	(47.8–63.9)
Emotional/social problems	55	38.5	(30.8–46.6)
Loss of follow up	26	18.2	(12.5–25.1)
Withdrawal symptoms	21	14.7	(9.6–21.2)
Economic problems	12	8.4	(4.7–13.8)
Unavailability of medication	6	4.2	(1.8–8.4)
Side effects of medications dispensed	5	3.5	(1.3–7.5)

As shown in Table 4, half of the relapses occurred early after quitting smoking during follow-up with smoking cessation clinics.

**Table 4.** Frequency distribution of relapse timing among the study sample.

Relapse timing	*N*	%
(1–6)	70	51.1
(7–12)	40	29.2
(13–24)	21	15.3
(>24)	6	4.4
Total	137	100.0

Six individuals had missing data for relapse timing.

All the tested sociodemographic variables, namely: age, sex, nationality, income, education, and marital status, failed to show a statistically significant association with early timing of relapse, Table 5.

**Table 5.** The association between selected sociodemographic variables and early relapse.

The variable	Relapse timing							*p*
	Later than 6 months		Early relapse (1–6 months)			Total		
	*N*	%	*N*	%	95% confidence interval %	*N*	%	
Age group (years)								0.21[NS]
<30	5	41.7	7	58.3	(31.2–82)	12	100.0	
30–39	29	47.5	32	52.5	(40.1–64.6)	61	100.0	
40–49	24	63.2	14	36.8	(22.9–52.7)	38	100.0	
50–59	7	38.9	11	61.1	(38.3–80.6)	18	100.0	
60+	2	25.0	6	75.0	(40.8–94.4)	8	100.0	
Gender								1[NS]
Female	3	42.9	4	57.1	(23.5–86.1)	7	100.0	
Male	64	49.2	66	50.8	(42.2–59.3)	130	100.0	
Nationality								0.69[NS]
Qataris	3	37.5	5	62.5	(29.5–88.1)	8	100.0	
Arabs (excluding Qataris)	56	48.7	59	51.3	(42.2–60.3)	115	100.0	
Others	8	57.1	6	42.9	(20.3–68.1)	14	100.0	
Average monthly income								0.49[NS]
<10,000	37	53.6	32	46.4	(35–58.1)	69	100.0	
10,000–19,999	19	46.3	22	53.7	(38.6–68.2)	41	100.0	
≥20,000	11	40.7	16	59.3	(40.6–76.1)	27	100.0	
Ever married								0.93[NS]
Single	8	50.0	8	50.0	(27.2–72.8)	16	100.0	
Ever married	59	48.8	62	51.2	(42.4–60)	121	100.0	
Educational level								0.69[NS]
Did not complete secondary school	13	50.0	13	50.0	(31.6–68.4)	26	100.0	
Completed secondary school	8	40.0	12	60.0	(38.4–78.9)	20	100.0	
Diploma/university education and above	46	50.5	45	49.5	(39.3–59.6)	91	100.0	

## Discussion

The SCSs are a key part of Qatar’s national primary health care, focused on addressing all types of tobacco use. As part of a larger project, this research examines the causes and the timing of smoking relapse and the prevalence of withdrawal symptoms that may contribute to it.^17^

The main findings of the study highlighted that relationships with smokers, emotional/social problems, and loss of follow-up were the most common reasons for smoking relapse. Additionally, an increase in appetite and weight gain followed by cravings for cigarettes/smoking represented the highest prevalence of smoking withdrawal symptoms. In terms of smoking relapses timing, early relapsing was common as 51.1% of relapsed cases occurred within 1–6 months after quitting, while 29.2% relapsed between 7 and 12 months following cessation.

Studies indicated that smoking relapse should not be considered as a failure, but as a moment of reflection on the factors that made them restart, and better prepare them for the next attempt, since most smokers try to stop three to four times on average until they definitively cease.^35^

The study reported that relationship with other smokers was one of the main reasons for smoking relapse and initiation which is substantiated with similar findings reported in published literature pertaining to smoking cessation and relapse.^21,36–38^ Similarly a study conducted in Australia reported that the likelihood of smoking relapse was increased in case of living with another smoker.^22^ Residing in environments where indoor smoking is prohibited is associated with a 60% reduction in the likelihood of relapse, compared to living in homes where smoking is permitted.^18^ Evidence also suggests that influence of surrounding smokers or smoking environments are one of the main barriers to quit smoking.^39^ Moreover, literature also suggests that a high level of engagement with smokers socially is associated with failure in long-term abstinence.^24,40^

The study reports that the social and emotional determinants play an important role and can pose as an important barrier to smoking cessation.^23,24^ Similarly a study reported that family pressure as one of the main reason for smoking relapsing among 30% of relapsed smokers, and social situations represented a main triggering factor among 35% of them.^31^ Additionally, exposure to stressful situations and lack of family support are often reported reasons for failure in smoking cessation.^21^ Findings of a study revealed that a little portion of relapsed smokers reported feeling negative or down and times of being alone as triggers for smoking relapse.^31^

A significant percentage of participants (18.2%) reported loss of follow-up with the provider of SCSs as a reason for relapsing. One of the main components of SCCs is following up smokers through phone calls and setting up appointments to provide sustained support, encouragement and necessary instructions to continue quitting smoking. Therefore, losing follow-up is sometimes a major reason for returning to smoking. In a study from Turkey, the number of follow-up calls was an important factor in maintaining the non-smoking status, as the number of calls was significantly higher in the non-relapsed group compared to the relapsed one.^33^

There were different smoking withdrawal symptoms associated with smoking cessation which are reported by participants who quit smoking in the study. More than 14% of participants stated that having one or more of withdrawal symptoms were the reason behind their relapse and failure in continuing the non-smoking status.^24,41^ The results from a study showed that there is a temporal relationship between withdrawal symptoms and the smoking relapse. Whereas higher negative affect, craving and withdrawal symptoms increased the likelihood of subsequent smoking relapse, and that smoking relapse led to subsequent increases in these same symptoms.^42^ In the first week, withdrawal symptoms are the most intense.^43^ Fatigue and increased tension were reported in all of patients throughout the first week of a study conducted in Turkey.^44^ In a study from China, withdrawal symptoms represented a main reason for smoking relapse.^24^

About three-fourths of smokers who had quit smoking reported having an increase in appetite and weight gain. This finding is substantiated with the findings of other studies which reported increase in the BMI and the HbA1c after 3 months smoking cessation.^45–47^ On the contrary, only 2.8% reported a drop in their weight.^48^ These findings substantiate with the fact that a strategic plan to address appetite and nutrition changes is of extreme importance during smoking cessation progression. Literature suggests exercise and the quality of diet as important indicators in this context.^49^ A randomized clinical trial conducted on female smokers documented that intervention group (regular exercise) compared to control ones (no exercise) had significantly higher levels of continuous abstinence, functional capacity had gained less weight by the end of treatment.^50^

Craving for smoking represented a main cause for relapsing in this study and others, as well as the level of craving was affecting the relapse where smokers with high craving had higher rate of relapse compared to smokers of low craving (*p* < 0.001).^25–27^

A significant percentage of those who have quit smoking in this study reported changes in their psychological state which led to their relapsing. These changes included feeling of anger, insomnia, difficulties in concentration, feeling of frustration, and depression. The results of this study are supported by various studies from different parts of the world.^26,27,45^ On the contrary, evidence indicates that cigarettes contain nicotine, a stimulant increasing the risk of insomnia which can lead to shorter sleep duration and cause irritability and restlessness. A study showed that night-time smoking was significantly associated with greater insomnia and shorter sleep duration.^29^ Also, initiating sleep is difficult among smokers compared to nonsmokers.^51^ Several studies highlighted that smoking cessation was associated with improving in psychological status of quitters. This positive effect led to improving in depression symptoms, decreasing in anxiety, stress levels, improving the quality of life, improving the positive mood, and reduced using the mental health medicines.^52,53^

The study also reported economic problems as a reason for smoking relapse by few participants in the study. On the contrary, this factor was significantly reported in other studies.^31,54,55^ A study suggested that when pre-quit financial problem is significant, mostly the post-quit withdrawal symptom severity would be greater, which In-turn increased the likelihood of relapsing after the scheduled quit attempt.^56^ Unavailability of medications or their side effects were identified as factors contributing to smoking relapse in this and other studies.^32,33^

The association between selected sociodemographic variables and early smoking relapse was not statistically significant in this study; however, early relapse remained the most common timing for smoking relapse. Previous studies found that most smoking relapses occurred soon after quitting, with nearly 75% of smokers relapsing within 6 months as indicated by this study.^13,15^ Another study indicated that the initial 2 years after quitting associated with higher relapse rates.^38^ Moreover, the likelihood of relapse decreases with extended abstinence as indicated in other studies.^16,17,38^ In this context, a longer period of abstinence predicts a lower relapse rate among quitters.^15–17^ Furthermore, findings from a separate study indicated that cessation of smoking for 6 months or longer was a significant predictor of sustained abstinence, even after accounting for additional variables. Individuals who reported abstaining from smoking for more than 6 months exhibited an 87% reduction in the odds of relapse compared to those who had quit for less than 6 months.^18^ Sensitivity analyses within the same research, employing various cut-off points for the duration of cessation prior to the baseline survey, consistently demonstrated that extended periods of prior abstinence were strongly correlated with reduced odds of relapse.^18^

This study demonstrated that the relationship between selected sociodemographic factors and smoking relapse varies depending on the specific factor, as evidenced by comparisons with prior research. Regarding the impact of age on smoking relapse, findings of this study suggested that increasing age correlates with a higher risk of relapse. Specifically, relapse rates are greatest among individuals over 50 years old, lower in those under 40, and lowest among those aged 40–49. This trend may relate to longer smoking histories in older adults. However, other research has shown that younger individuals can also exhibit elevated relapse rates.^57,58^ One study found that participants aged ≥ 45 years tended to maintain abstinence for longer durations,^24^ while another indicated that advanced age is linked to a decreased likelihood of relapse.^38^

This study found higher relapse rates among female quitters, though other research reported lower rates for women.^57^ In this context, another study indicated that psychological factors like emotion and stress mainly influence women’s relapse, while environmental factors are more significant for men.^59^

The relation between educational level and smoking relapse in this study showed that those with middle educational attainment had higher relapse rates than individuals at higher or lower levels, aligning with previous findings.^57^ One study found longer cessation among those with low education,^24^ while another reported that higher education decreased relapse risk.^38^

The relapse rates in this study showed minimal variation between individuals who had ever been married and those who had never married. A study indicated that in-marriage or unmarried individuals have higher relapse rates than divorced or widowed people.^57^ Compared to married individuals, former smokers who were widowed or divorced demonstrated a 2.77 and 2.34 times greater likelihood of relapse, respectively, while those who were separated exhibited approximately fourfold increased odds of relapse.^18^

Regarding the association of smoking relapses with the smokers’ income in this study, higher income was linked to more relapses, opposing previous findings.^57,60^ Another study found that financial strain in adult smokers correlates with lower quit rates and higher relapse rates.^60^

## Conclusion

The study highlighted multifaceted factors associated with smoking relapses among smokers accessing smoking cessation services. Relationships with smokers and emotional or social problems were the most common reasons. Early relapses represent the main challenges facing smoking cessation initiatives. Future research on smoking cessation interventions should prioritize the initial months of abstinence to address early instances of smoking relapse.

## Recommendations

- Smoking relapse is a significant issue for anti-smoking efforts locally and globally. These initiatives often require substantial human and financial resources, particularly in countries like Qatar that offer free services. Implementing evidence-based strategies and emphasizing research are crucial for preventing smoking relapses.
- Developing a smoking relapse prevention program for at least 2 years is essential, as relapse rates decrease with prolonged abstinence.
- Targeting withdrawal symptoms may reduce smoking relapses.

## Strengths of the study

- The follow-up duration extended to 42 months represents an advantage to detect the best prevalence of smoking relapse and the socio-demographic impacts of smoking cessation.
- The ability to examine multiple outcomes over long time duration.

## Limitations of the Study

- The primary limitations of this study are potential sample biases and reliance on self-reports. Self-reported data, especially after extended periods, may introduce recall bias, leading to inaccurate outcomes and possible overestimation of prevalence.
- The cross-sectional analysis of a moderate size sample (143) may reduce the external validity of the study conclusions. However, the results are valuable given the lack of similar studies on smoking cessation and relapses locally.

## Supplemental Material

sj-docx-1-phj-10.1177_22799036251401951 – Supplemental material for Smoking relapse and withdrawal symptoms among smokers accessing smoking cessation services provided by the primary care settings of Qatar Supplemental material, sj-docx-1-phj-10.1177_22799036251401951 for Smoking relapse and withdrawal symptoms among smokers accessing smoking cessation services provided by the primary care settings of Qatar by Abduljaleel Abdullatif Zainel, Hanan Al Mujalli, Ameena Ibrahim Yfakhroo, Shaikha Abdulla Shaheen, Hafiz Ahmed E. Mohamed, Ahmed Sameer Al Nuaimi, Muslim Abbas Syed and Mohamed Ahmed Syed in Journal of Public Health Research
